# Biomechanical analysis of contact pressure generated by the calcaneofibular ligament against the peroneus brevis tendon: A cadaveric study

**DOI:** 10.1002/jeo2.70330

**Published:** 2025-07-13

**Authors:** Hisayoshi Yoshizuka, Yutaro Nakao, Akio Kuraoka

**Affiliations:** ^1^ Department of Physical Therapy, Faculty of Medical Science Fukuoka International University of Health and Welfare Fukuoka Japan; ^2^ Department of Anatomy and Physiology, Faculty of Medicine Saga University Saga Japan; ^3^ Department of Rehabilitation Medicine Nishikawa Orthopedic Clinic Ogi Japan

**Keywords:** ankle, cadaver, calcaneofibular ligament, peroneus brevis tendon, pressure‐sensitive sensor

## Abstract

**Purpose:**

A recent cadaveric study revealed that the tensed calcaneofibular ligament (CFL) lifts the peroneal tendons. This suggests that the CFL acts as a tensioner to transmit the contractile activity of the peroneal muscles effectively. However, the onset points of the lift‐up and actual CFL‐generated contact pressures against the peroneus brevis tendon (PBT) remain unclear. This cadaveric study aimed to elucidate the lift‐up phenomenon through the quantitative measurement of changes in the contact pressure between the CFL and PBT under precise monitoring of the ankle position.

**Methods:**

We performed a quantitative analysis of 11 cadaveric ankle specimens with a pressure‐sensitive sensor under accurate three‐axial joint angle monitoring using inertial measurement units. The output values from the pressure‐sensitive sensor inserted between the CFL and PBT were measured at different inversion (INV) angles, converted into contact pressure values (mV), and statistically analysed.

**Results:**

Although the onset points differed across the samples, the contact pressure values of all samples showed a positive change as the INV angle increased. Friedman and post hoc Steel–Dwass tests revealed significant differences between INV 0° versus 10° (*p* < 0.01), 0° versus 15° (*p* < 0.001), 5° versus 10° (*p* < 0.05), 5° versus 15° (*p* < 0.001), and 10° versus 15° (*p* < 0.05). In addition, the sensor output values indicated an intraclass correlation coefficient (1, 1) of 0.90.

**Conclusion:**

Our findings strongly suggest that INV enhances the tensioner effect of CFL. Specifically, an ankle with a relatively early onset point and high contact pressure may exhibit a superior tensioner effect and, through the control of peroneal tendons, potentially prevent an ankle sprain.

**Level of Evidence:**

Not applicable.

**Abbreviations**
CFL: calcaneofibular ligament
CPV: contact pressure value
DF: dorsiflexion
ICC: intraclass correlation coefficient
IMU: inertial measurement unit
INV: inversion
PBT: peroneus brevis tendon
PF: plantarflexion
PLT: peroneus longus tendon
PSS: pressure‐sensitive sensor

## INTRODUCTION

The calcaneofibular ligament (CFL), part of the lateral ligament complex of the ankle along with the anterior and posterior talofibular ligaments, tightens during dorsiflexion (DF) [19, 21, 26] or inversion (INV) [4, 9, 16, 25, 26] and plays a key role in stabilising the ankle joint [10].

**Figure 1:**
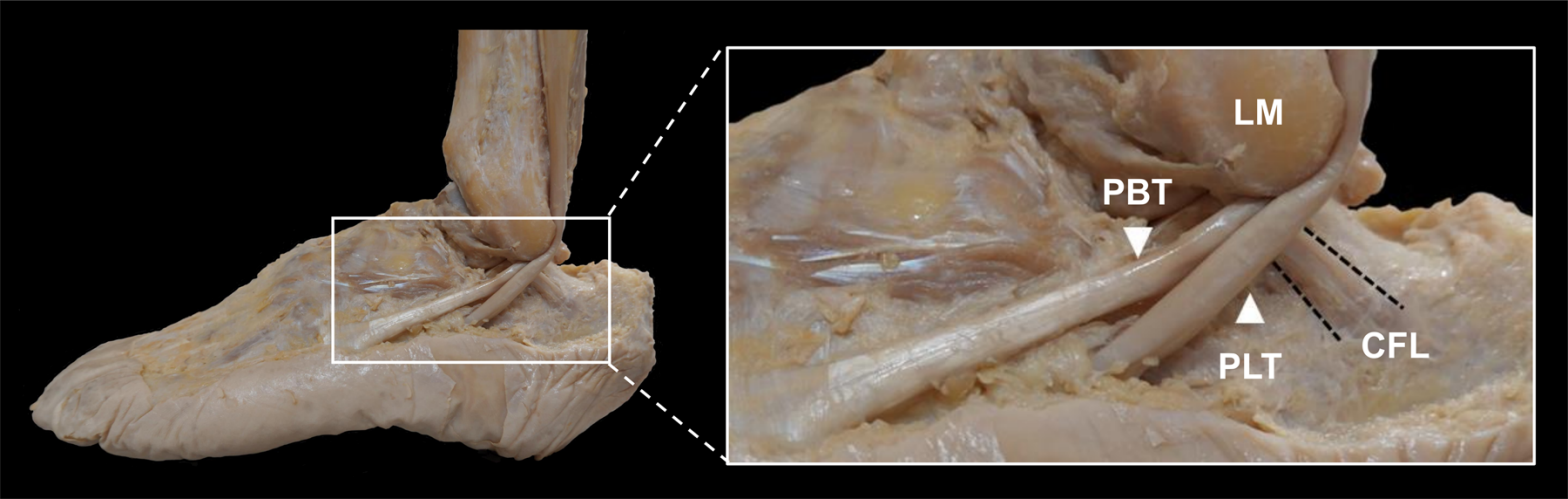
Positional relationship between the peroneus longus tendon (PLT), peroneus brevis tendon (PBT), and calcaneofibular ligament (CFL) in a left cadaveric ankle. The dashed black line indicates the margin of the CFL. LM, lateral malleolus.

Below the lateral malleolus, the CFL crosses closely beneath the deep side of the peroneus brevis tendon (PBT) [27] (Figure 1). A recent cadaveric study on this close proximity found that the tensed CFL significantly lifts the peroneus longus tendon (PLT) and PBT laterally, as shown by coordinate analysis using a contactless three‐dimensional scanning system [25]. This lift suggests that the CFL acts as a tensioner for the effective transmission of the peroneal muscle contraction and contributes to ankle stability by modifying the mechanical behaviour of the peroneal tendons, particularly when walking on uneven terrain. However, this verification was limited to coordinate analysis at only one position (15° INV with 10° plantarflexion [PF]). Therefore, the onset points of the lift‐up and the actual contact pressures generated by the CFL remain unclear.

Conventionally, cadaveric functional analyses are conducted on specimens fixed to an apparatus, with their limb positions adjusted and measured using a goniometric method. However, the ankle moves in a complex manner along three axes: PF/DF, INV/eversion, and abduction/adduction [3]. Previous studies on the reliability of ankle motion range measurements have indicated a lack of excellent reproducibility, even in a single direction, when using a goniometer [2, 5].

Pressure‐sensitive sensors (PSS) have been used to measure joint contact pressure in cadaveric ankles [11, 13]. Recently developed ultra‐thin and small‐sized PSS can detect minute pressures, making them well‐suited to accurately assess changes in contact pressure between soft tissues, such as tendons and ligaments. Additionally, a recent cadaveric study monitored ankle joint angles along three axes using an inertial measurement unit (IMU) [26], producing highly reproducible experimental results (intraclass correlation coefficient [ICC] [1.1]: 0.89).

Using PSS and IMU, this cadaveric study aimed to elucidate the lift‐up phenomenon by quantitatively measuring changes in the contact pressure between the CFL and PBT while precisely monitoring the ankle position.

## MATERIALS AND METHODS

### Sample preparation

We assessed 18 ankles from nine formalin‐fixed Japanese cadavers (eight men and one woman) that had been used for a gross anatomy course at our university in 2022. The exclusion criteria were evidence of previous injury or surgical treatment in the ankle region, severe deformity of the plantar skin, and a significant reduction in the extensibility of the peroneal muscles. Ultimately, we included 11 ankles from six cadavers (five men and one woman). Age at the time of death ranged from 74 to 89 years, with a mean age ± standard deviation of 81 ± 6 years.

During the gross anatomical course, the skin, subcutaneous tissue, and deep fascia of the lower legs and feet were removed, whereas the peroneus longus, brevis, and lateral and medial ligament complexes were preserved. After amputation of the lower legs at the knee joint, the PLT, PBT and CFL were exposed through careful dissection from the surrounding tissues, such as the peroneal tendon sheath and connective fibres between the CFL and anterior talofibular ligament [12]. To facilitate the necessary ankle joint movement for the experiments, the posterior talofibular ligament and medial ligament complex were partially removed.

The study design was approved by the local ethics committee of our university (Authorization No. R3–10). The authors confirm that all efforts were made to adhere to local and international ethical guidelines and laws regarding the use of human cadaveric donors in anatomical research, and this study was performed in line with the principles of the Declaration of Helsinki. Informed consent for the storage and use of cadavers for research purposes was obtained in advance from the donors and their relatives.

### Evaluation of actual contact pressure values using a PSS

**Figure 2:**
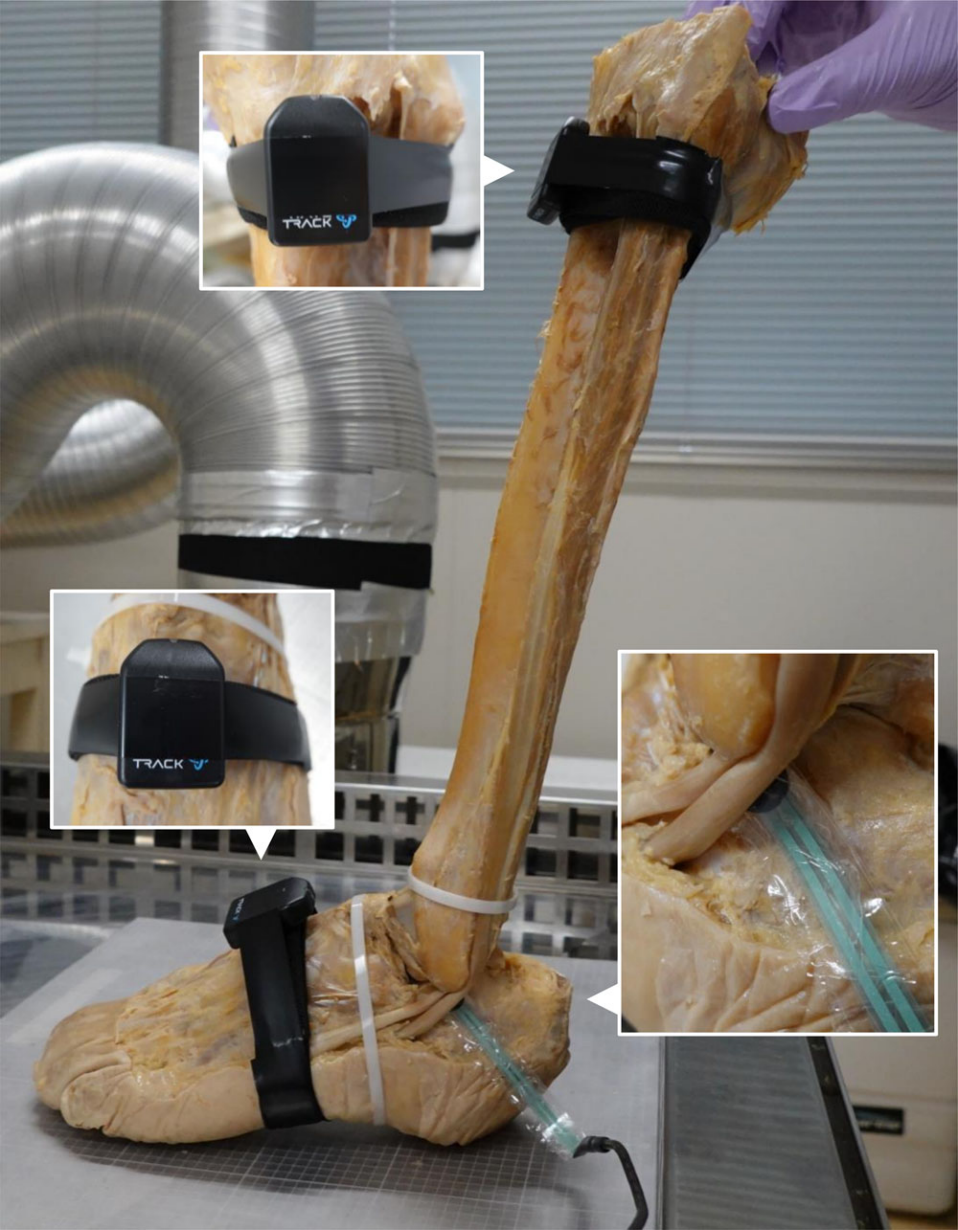
Placement of sensors. After attaching broad textile belts as mounting bases, two inertial sensors were positioned on the midfoot and superior portion of the anterior lower leg using adhesive tape (small insets). The pressure‐sensitive sensor, wrapped in a polyvinylidene chloride sheet, was placed between the peroneus brevis tendon and calcaneofibular ligament (large inset).

**Figure 3:**
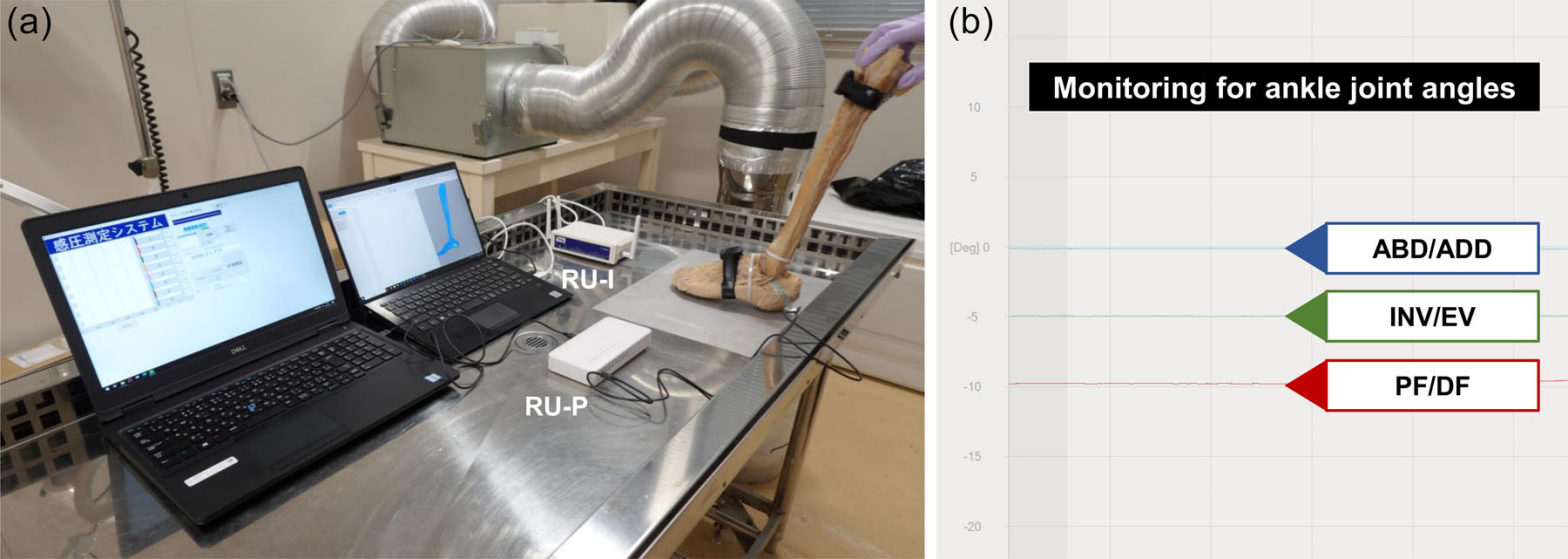
Overview of the measurement system. (a) RU‐I and RU‐P are the receiving units for the inertial measurement unit and pressure‐sensitive sensor, respectively. (b) During the experiments, the inertial measurement unit enabled visualisation of the ankle joint angles in three directions: abduction/adduction (ABD/ADD), inversion/eversion (INV/EV), and plantar/dorsiflexion (PF/DF).

Before the examination, the samples were secured with two plastic bands at the positions of the superior and inferior fibular retinacula to maintain the PLT and PBT loosely at their original sites (Figure 2). For the quantitative evaluation of the actual contact pressure, a circular‐shaped PSS for micro‐pressure measurement (diameter: 10 mm, thickness: 0.21 mm) (CKS10L‐F, Canon Chemicals, Ibaraki, Japan) [24] was wrapped in a polyvinylidene chloride sheet and inserted between the CFL and PBT (Figure 2). PSS measurement values were collected at a frequency of 64 Hz through a receiving unit (Figure 3a). The voltage value (mV), which indicates the actual contact pressure, was calculated as follows:

$$\mathrm{Voltage}\;\mathrm{value}\;\left(\mathrm{mV}\right)=\left(\frac{\mathrm{Sensor}\;\mathrm{output}\;\mathrm{value}}{1024}\right)\times 3.3\times 1000.$$

The PSS was calibrated for each specimen. Under stable conditions of 10° PF and 0° abduction/adduction, measurements were performed at 0°, 5°, 10°, and 15° INV, with each measurement lasting ≥5 s. The average of the calculated voltage values (320 data points for each INV angle) is designated as the contact pressure value (CPV).

### Method for monitoring ankle joint angles

To accurately monitor the three‐axis ankle joint angles, an IMU was used on the cadaveric specimens [26]. After attaching broad textile belts as mounting bases, two inertial sensors (WaveTrack, Cometa srl, Milan, Italy) were positioned on the midfoot and superior portion of the anterior lower leg, following the manufacturer's instructions (Figure 2). The IMU comprises inertial sensors and a receiving unit (Figure 3a). Real‐time ankle joint angles were visualised in three directions: DF/PF, INV/eversion, and abduction/adduction [3] (Figure 3b).

### Measurement methods for the CFL morphology

The length, width, and running angle of the CFL were measured using the talocrural and subtalar joints in the 0° position. Length was defined as the distance between the fibular origin and the most proximal insertion on the calcaneus [18, 25, 27]. The width was measured at the midpoint of the ligament [22, 23, 25]. In addition, we evaluated the CFL running angle, defined by the long axes of the CFL and fibula [20]. The fibular long axis was determined by connecting the two midpoint sets obtained by measuring the width of any region in the diaphysis [25, 27]. The accuracy of this technique was confirmed by performing the procedure in triplicate.

### Statistical analysis

All statistical analyses were conducted using R software version 4.3.0 (R Foundation for Statistical Computing, Vienna, Austria). Statistical significance was set at *p* < 0.05. The normality of the distribution of the collected data was assessed using the Shapiro–Wilk test. The ICC was used to measure test‐retest reliability and was interpreted as follows: poor (<0.50), moderate (0.50–0.74), good (0.75–0.90) and excellent (>0.90) [15]. The CPVs at different INV positions were assessed using the Friedman test. Post hoc pairwise comparisons were performed using the Steel–Dwass test. To examine the relationship between CPV and CFL morphology (length, width, and running angle), Pearson's or Spearman's correlation coefficients were calculated. Post hoc power analysis (1 − β) was performed using G*power version 3.1.9.7 (University of Düsseldorf, Düsseldorf, Germany) [7, 8] to evaluate whether our data had sufficient power.

## RESULTS

**Figure 4:**
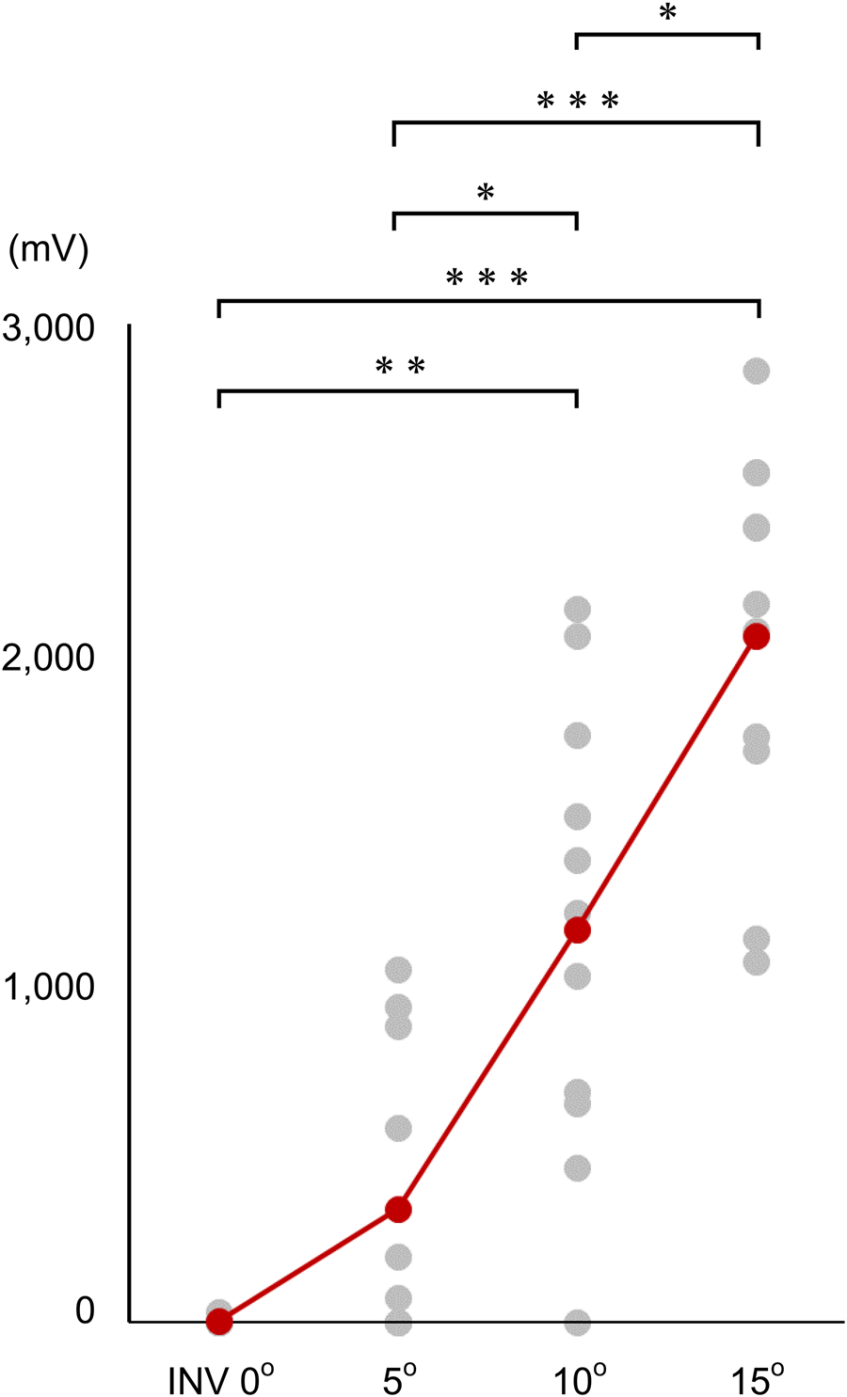
Results of post hoc pairwise comparison of CPV. The CPV (mV) was evaluated under 4 conditions: 10° PF with 0° INV/eversion, 10° PF with 5° INV, 10° PF with 10° INV, and 10° PF with 15° INV. The red and grey circles show the mean CPV and CPV of each specimen, respectively. CPV, contact pressure value; INV, inversion; PF, plantarflexion. *p < 0.05, **p < 0.01, ***p < 0.001.

Our findings revealed that the CPV of all samples showed a positive change as the INV angle increased (Supporting Information: Table S1). The results of the mean CPV are summarised in Table 1. The Friedman test revealed a significant difference (*p* < 0.001), with post hoc power analysis confirming >99% statistical power. The Steel–Dwass post hoc test revealed significant differences between INV 0° versus 10° (*p* < 0.01), 0° versus 15° (*p* < 0.001), 5° versus 10° (*p* < 0.05), 5° versus 15° (*p* < 0.001), and 10° versus 15° (*p* < 0.05) (Figure 4).

**Table 1:** Changes of the CPV.

	10° Plantar flexion with				
	0° INV	5° INV	10° INV	15° INV	Friedman Test
CPV (mV)	3.1 ± 9.3	340.9 ± 437.8	1178.6 ± 682.0	2063.0 ± 577.7	*p* < 0.001

*Note*: The values are presented as the mean ± standard deviation.

Abbreviations: CPV, contact pressure value; INV, inversion.

**Figure 5:**
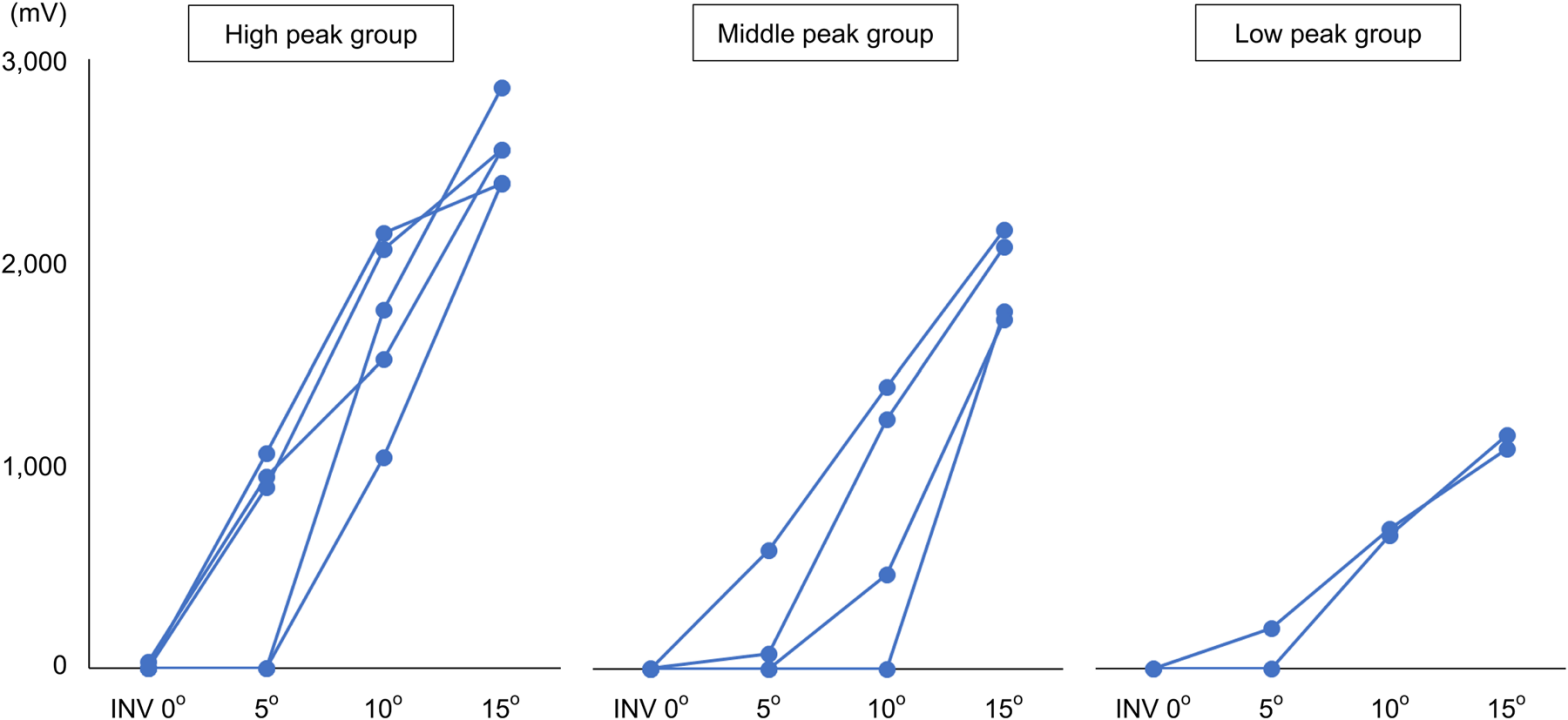
Changing patterns of the CPV in each sample. The peak CPV (mV) was roughly classified into three groups: high (*n* = 5), middle (*n* = 4), and low values (*n* = 2). CPV, contact pressure value; INV, inversion.

Regarding the behaviour of each sample, the trends in the changes differed (Figure 5 and Supporting Information: Table S1). Some cases exhibited a steep upward trend, whereas others showed a gentler trend. The onsets for 10 of the 11 cases occurred under an INV of 10°, with six of them between INV 0° and 5°. No relationship was observed between the trend patterns and onset. In the 10 paired samples, the mean CPV of the right side consistently exceeded that of the left side at each INV angle examined (data not shown). However, comparisons within the same individual revealed that the right side was not always dominant, and notably, the trend patterns were not the same (Supporting Information: Table S1).

The mean length, width, and running angle of the CFL were 16.3 ± 3.2 mm (range: 12.0–21.5 mm), 4.8 ± 1.0 mm (range: 3.5–6.5 mm) and 42.3° ± 12.9° (range: 15°–55°), respectively. No significant correlations were found between these measurements and CPV.

In the three specimens, after completing the first set of measurements, all sensors were removed and re‐installed, followed by a second round of measurements. The ICC (1, 1) for the sensor output values was 0.9003, indicating ‘excellent’ reliability and reproducibility [15].

## DISCUSSION

The principal finding of this study is that the contact pressure significantly increases with an increase in the INV angle, strongly suggesting that the INV enhances the tensioning effect of the CFL. Although we did not examine the effects on the PLT, which does not directly contact the CFL, a previous study using coordinate analysis on cadaveric specimens confirmed the synchronous movement of the PBT and PLT during the lift‐up phenomenon [25]. Therefore, pressure may also affect PLT. Additionally, the use of the IMU, which enables precise joint angle monitoring, demonstrated high reproducibility, which is consistent with previous reports [26].

The dimensional values of the CFL in our study were approximately within the ranges reported in previous cadaveric studies using the same measurement method: 10.0–30.0 mm for length [18, 25, 27], 4.0–6.0 mm for width [22, 23, 25] and 0°–90° for running angle [20]. Ligaments with atypical morphologies, including fan‐shaped CFLs [20] and those composed of two or three crossing fibre bundles [4, 14], were not included. Therefore, our samples were morphologically unbiased.

There were some inter‐ and intra‐individual variations in the level of the actual contact pressure and onset point. While it is important to identify the factors influencing these variations, no significant correlations were found between pressure and routinely evaluated CFL dimensions. Therefore, other parameters are likely to be involved. For example, variations in the fibular origin and calcaneal insertion sites of the CFL [17] could affect the actual contact pressure or onset.

To relax stiff peroneal tendons in formalin‐fixed cadavers, a previous functional analysis of the CFL maintained 10° PF throughout the experiment [25], similar to our study. A recent functional analysis of cadaveric CFL using stretchable strain sensors found that the combination of INV and DF generated the highest tension, surpassing that of INV or DF [26]. Therefore, it is reasonable to speculate that there may be ankle positions other than those examined in this study where the tensioning effect is maximised. Future in vitro studies using fresh or Thiel‐embalmed cadavers or in vivo studies using non‐invasive modalities, such as ultrasound, are needed to further elucidate the full extent of the lift‐up phenomenon.

The peroneus longus and brevis muscles are crucial for mediolateral stability during human bipedal locomotion and for preventing unintended ankle INV [1]. Based on our findings, an ankle with early onset and high contact pressure may exhibit a superior tensioning effect, potentially aiding in the control of the PBT and PLT to prevent sprains. Another hypothesis is that an excessive lift‐up phenomenon due to extremely high contact pressure could be associated with the mechanism of peroneal tendon subluxation and dislocation [6]. Taken together, these insights offer a new perspective for understanding ankle joint function and pathophysiology.

### Limitations

This study had several limitations. First, the subjects were very old, and the lack of detailed records regarding sprains or chronic ankle instability during their lifetime may have impacted the accuracy of our findings. Second, although post‐hoc power analyses indicated that our data had sufficient statistical power, the sample size was relatively small owing to the stringent exclusion criteria. Third, during sample preparation, partial removal of the posterior talofibular ligament and the medial ligament complex was necessary to ensure ankle joint mobility in formalin‐fixed cadavers. Additionally, the superior and inferior fibular retinacula and the peroneal tendon sheath were removed, and the former was replaced with two plastic bands [25] to insert the PSS between the CFL and PBT. These factors may have influenced our findings. Fourth, the use of PSS wrapped in a thin polyvinylidene chloride sheet may have influenced the measurements; however, this wrapping was necessary to protect the PSS in formalin‐fixed cadavers, as it is not water‐resistant.

## CONCLUSION

Our findings demonstrated that the contact pressure significantly increased with an increase in the INV angle, strongly suggesting that the INV enhances the tensioning effect of the CFL on the PBT and PLT. Additionally, the use of the IMU, which allows precise joint angle monitoring, showed high reproducibility and is expected to be valuable in further biomechanical studies on cadavers.

## AUTHOR CONTRIBUTIONS

This study was jointly designed by Hisayoshi Yoshizuka, Yutaro Nakao and Akio Kuraoka. The data were collected by Hisayoshi Yoshizuka and Yutaro Nakao. Data analysis was performed by Hisayoshi Yoshizuka. The manuscript was written by Hisayoshi Yoshizuka and Akio Kuraoka. All authors approved the final version of the manuscript.

## CONFLICT OF INTEREST STATEMENT

The authors declare no conflicts of interest.

## ETHICS STATEMENT

The study design was approved by the local ethics committee of Saga University (authorization number R3‐10). Informed consent for the storage and use of the cadavers for research purposes was provided in advance by the donors and their relatives. All methods were performed in accordance with the relevant guidelines and regulations.

## Supporting information

Supporting Information.

## Data Availability

The data that support the findings of this study are available from the corresponding author upon reasonable request.
