# Supplementary material for: Biomechanical analysis of contact pressure generated by the calcaneofibular ligament against the peroneus brevis tendon: A cadaveric study
Source: J Exp Orthop. 2025 Jul 13;12(3):e70330. doi: 10.1002/jeo2.70330 (PMC12255950; doi:10.1002/jeo2.70330)
Supplement: Supplementary file 1 — Supporting Information. [file JEO2-12-e70330-s001.docx]

***Journal of Experimental Orthopaedics***

**Supplementary Information for**

**Biomechanical analysis of contact pressure generated by the calcaneofibular ligament against the peroneus brevis tendon: A cadaveric study**

**Table S1** Measurement values in each sample

| No. | Age (year) | Sex | Side | Dimensions of the CFL | | |  | Contact pressure values (mV) | | | |
| --- | --- | --- | --- | --- | --- | --- | --- | --- | --- | --- | --- |
|  |  |  |  | Length  (mm) | Width  (mm) | Angle  (degree) |  | INV  0° | INV  5° | INV  10° | INV  15° |
| 1 | 74 | M | L | 21.5 | 6.5 | 15 |  | 0 | 0 | 1,764.2 | 2,858.9 |
| 2 | 85 | M | R | 20.0 | 5.0 | 50 |  | 0 | 1,058.4 | 2,144.8 | 2,388.5 |
| 3 |  |  | L | 19.0 | 5.0 | 45 |  | 0 | 0 | 656.9 | 1,151.8 |
| 4 | 80 | M | R | 16.0 | 5.0 | 55 |  | 0 | 0 | 463.5 | 1,718.8 |
| 5 |  |  | L | 19.0 | 6.5 | 50 |  | 0 | 891.3 | 2,064.0 | 2,555.4 |
| 6 | 81 | M | R | 15.0 | 3.5 | 40 |  | 30.9 | 946.6 | 1,520.7 | 2,553.9 |
| 7 |  |  | L | 15.0 | 4.5 | 30 |  | 3.7 | 72.7 | 1,229.2 | 2,078.3 |
| 8 | 75 | M | R | 16.0 | 4.5 | 55 |  | 0 | 0 | 1,042.0 | 2,392.4 |
| 9 |  |  | L | 14.0 | 5.0 | 55 |  | 0 | 0 | 0 | 1,759.5 |
| 10 | 89 | F | R | 12.0 | 3.5 | 40 |  | 0 | 198.5 | 692.0 | 1,085.4 |
| 11 |  |  | L | 12.0 | 4.0 | 30 |  | 0 | 582.7 | 1,387.7 | 2,159.0 |

CFL, calcaneofibular ligament; INV, inversion; M, male: F, Female
